# Supplementary material for: Consumers’ Attitude towards Sustainability in Italy: Process of Validation of a Duly Designed Questionnaire
Source: Foods. 2022 Aug 30;11(17):2629. doi: 10.3390/foods11172629 (PMC9455261; doi:10.3390/foods11172629)
Supplement: Supplementary file 1 [file foods-11-02629-s001.zip › Table S1.pdf]

Table S1: Food Sustainability Questionnaire (first version)

This is not a test to evaluate your diet but a survey. It is important that you answer individually, your questionnaire will be anonymous. Thank you for devoting time to this questionnaire.

1. To what extent do you agree with each of the following statements?
[answer from 1 (strongly disagree) to 10 (strongly agree)]

My food habits negatively affect the environment	
When compared to car use, food habits have only little impact on the environment	
In relative terms, the environmental impact resulting from food habits and food production in the European Union is smaller than it is in countries such as China or the USA	

2. How much attention do you pay to the impact ofyour food choices on the environment? <u>(Tick only one answer)</u>

0 = I don't care if my food choices affect the environment or not	
1 = I pay few attention	
2 = I pay some attention	
3 = I pay a lot of attention	

3. What comes to your mind when thinking about “sustainable” food? <u>(Tick maximum 3 items)</u>

Low environmental impact		
Availability and affordability of food for all		
Use of pesticides and GMOs to be avoided		
Local supply chains		
Fair revenue for farmers		
High animal welfare standards		
Economic growth in the agri-food sector		
Minimally processed, traditional		
Healthy		

4. To what extent would you say that your eating habits are influenced by sustainability concerns?

0 = no single influence	
1 = minor influence	
2 = some influence	
3 = big influence	
4 = I don’t know	

5. What are the main reasons preventing you from eating (more) sustainably? <u>(Tick maximum 3 reasons)</u>

Lack of information on how to do so	
Lack of clear labelling	
I’m not concerned with sustainability	
Lack of sustainable food products in my usual shopping places / eating places	
Too expensive	
I’m not willing to change my eating habits	
Lack of time (to buy it, to cook it, etc.)	

Other reason	

6. To what extent do you agree with each of the following statements?
[answer from 1 (strongly disagree) to 10 (strongly agree)]

I’m willing to buy mainly seasonal fruit and vegetables	
I’m willing to spend more money for sustainable food	
I’m willing to spend more money on food for which I’m sure that farmers get a fair price in return	
I’m willing to cut down on red meat (beef, lamb and pork)	
I’m willing to cut down on dairy	
I am willing to waste less food at home	
I'm willing to eat more vegetables/plant-based food	
I’m not willing to change my eating habits, even if they are not environment-friendly	

7. Have you reduced (o r do you intend to reduce) your red meat (beef, lamb and pork) consumpotion due to environmental reasons? (Tick only one answer)

I don't eat meat, because I'm vegetarian/vegan	
Yes, I’ve stopped eating red meat for environmental reasons (though I’m not vegetarian/vegan)	
Yes, I’ve reduced the consumption of red meat (but still eat it)	
Yes, I’m intending to reduce the consumption of red meat for environmental reasons	
Yes, I'm intending to stop eating red meat for environmental reasons	
No, I didn’t reduce red meat consumption, nor do I intend to do it due to environmental reasons	

8. With which protein source do you replace or would you preferably replace meat at meals? (Tick maximum 3 reasons)

Fish		
Eggs		
Dairy products		
Protein powder (Instant meat substitutes)		
Legumes		
Nuts		
Seitan		
Tempeh		
Algae		
Jellyfish		

9. In the future, would you be willing to replace meat with each of the following food items?

	YES	NO	I DO NOT KNOW
Insects and insect derivates			
Lab-grown meat (from cell culture)			
Plant-based meat alternatives, only made from ingredients that are not derived from Genetically Modified Organisms (GMO)			
Plant-based meat alternatives, even if made from ingredients derived from Genetically Modified Organisms (GMO)			

Traditional vegetarian foods (e.g. vegetable stew)			
Algae/jellyfish			

10. To what extent do you agree with each of the following statements?
 [answer from 1 (strongly disagree) to 10 (strongly agree)

Eating meat is necessary to have a complete diet	
I need meat to have energy	
Eating meat allows me to have a balanced diet	
Meat is irreplaceable in my diet	
It is possible to have a balanced diet even without the consumption of meat	
Replacing meat with plant-based protein sources doesn't provide me with the same energy	

11. If all meat products comply with farm animal welfare rules and prices were to increase by 30% compared to current prices (<u>Tick only one answer</u>):

I would eat the same amount of meat products as I currently do	
I would eat more meat products than I currently do	
I would eat less meat products than I currently do	

12. To what extent do you agree that companies use meat-related names like 'sausage' and 'burger' to describe meat-free vegetarian products (e.g. a veggie 'burger')? <u>(Tick only one answer):</u>

It should never be allowed for vegetarian products	
It should be allowed only if it is clearly labelled it's a vegetarian product	
I don't see any problem for using such names	
I have no opinion	

13. To what extent do you agree with each of the following statements?
 [answer from 1 (strongly disagree) to 10 (strongly agree)]

Sustainability information should be compulsory on food labels	
Food which is less sustainable should be more taxed (and be more expensive)	
Unsustainable food products should be pulled from shelves (e.g. no strawberries in winter, supermarkets should only sell fish sourced sustainably, etc.)	
I do not want someone to tell me or decide for me what I should eat or not	
Regulations should force farmers and food producers to meet more stringent sustainability standards (in terms of greenhouse gas emissions, water use, biodiversity impact, etc.)	
Farmers should be given incentives (e.g. through subsidies) to produce food more sustainably	
The EU should not be more proactive on sustainable food policies unless other countries such as China or the USA do the same	
The government is doing enough in encouraging/promoting food sustainability (e.g. public campaigns, incentives)	

14. To what extent do the following activities contribute to climate change?
 [answer from 1 (strongly disagree) to 10 (strongly agree)]

Gas emissions from aircraft, trains, cars, trucks and ships	
The production of meat and dairy products, which we eat and drink	

The felling of trees and forests	
