# Supplementary material for: Consumers’ Attitude towards Sustainability in Italy: Process of Validation of a Duly Designed Questionnaire
Source: Foods. 2022 Aug 30;11(17):2629. doi: 10.3390/foods11172629 (PMC9455261; doi:10.3390/foods11172629)
Supplement: Supplementary file 1 [file foods-11-02629-s001.zip › Table S2.pdf]

Table S2 Socio-demographic characteristics of the pilot group.

Variables	Participants (n=150)
**Gender**	
Woman	74.6%
Man	24,6%
Prefer no answer	0.6%
**Age (years)**	
18-29	27,3%
30-49	34,0%
50-69	35,3%
70-79	1,3%
>80	0,7%
**Anthropometric measurements**	
Height (cm)	168.43(8.6)
Weight (kg)	67.41(13.5)
BMI (kg/m²)	23.70(4.1)
**Education Level**	
University or more	74%
Primary and secondary high school	26%
**Family income (euros)**	
<18.000	13,3%
18.001 and 27.000	22,0%
27.001 and 36.000	11,3%
36.001 and 54.000	14,7%
54.001 and 72.000	6,0%
>72.000	6,7%
Prefer no answer	26,0%
