# Supplementary material for: Consumers’ Attitude towards Sustainability in Italy: Process of Validation of a Duly Designed Questionnaire
Source: Foods. 2022 Aug 30;11(17):2629. doi: 10.3390/foods11172629 (PMC9455261; doi:10.3390/foods11172629)
Supplement: Supplementary file 1 [file foods-11-02629-s001.zip › Table S3.pdf]

# Table S3: Food Sustainability Questionnaire (final version)

*This is not a test to evaluate your diet but a survey. It is important that you answer individually, your questionnaire will be anonymous. Thank you for devoting time to this questionnaire.*

1. To what extent do you agree with each of the following statements?
   [answer from 1 (strongly disagree) to 10 (strongly agree)]

| | |
|---|---|
| My food habits negatively affect the environment | |
| When compared to car use, food habits have only little impact on the environment | |
| I pay attention to the impact that my food choices have on the environment. | |
| Sustainability issues influence my food choices. | |
| I improved my habits in favor of food sustainability, following the government's advertising campaigns. | |
| I do not want someone to tell me or decide for me what I should eat or not | |

2. What comes to your mind when thinking about "sustainable" food?

| | YES | NO |
|---|---|---|
| Low environmental impact | | |
| Availability and affordability of food for all | | |
| Use of pesticides and GMOs to be avoided | | |
| Local supply chains | | |
| Fair revenue for farmers | | |
| High animal welfare standards | | |
| Economic growth in the agri-food sector | | |
| Minimally processed, traditional | | |
| Healthy | | |

3. What are the main reasons preventing you from eating (more) sustainably?

| | YES | NO |
|---|---|---|
| Lack of information on how to do so | | |
| Lack of clear labelling | | |
| I'm not concerned with sustainability | | |
| Lack of sustainable food products in my usual shopping places / eating places | | |
| Too expensive | | |
| I'm not willing to change my eating habits | | |
| Lack of time (to buy it, to cook it, etc.) | | |

4. To what extent do you agree with each of the following statements?
   [answer from 1 (strongly disagree) to 10 (strongly agree)]

| | |
|---|---|
| I'm willing to buy mainly seasonal fruit and vegetables | |
| I'm willing to spend more money for sustainable food | |
| I'm willing to spend more money on food for which I'm sure that farmers get a fair price in return | |
| I'm willing to cut down on red meat (beef, lamb and pork) | |
| I'm willing to cut down on dairy | |
| I am willing to waste less food at home, implementing anti-waste measures (e.g. shopping list, placing foods that expire first in front of the refrigerator, etc. | |

| | |
|---|---|
| I'm willing to eat more vegetables/plant-based food, even if they're not to my taste | |
| I'm willing to change my eating habits, even if they are not environment-friendly | |

5. Have you reduced (or plans to reduce) the consumption of red meat (beef, lamb and pork) for environmental reasons? (Tick only one answer)

| | |
|---|---|
| I don't eat meat, because I'm vegetarian/vegan | |
| Yes, I've stopped eating red meat for environmental reasons (though I'm not vegetarian/vegan) | |
| Yes, I've reduced the consumption of red meat (but still eat it) | |
| Yes, I'm intending to reduce the consumption of red meat for environmental reasons | |
| Yes, I'm intending to stop eating red meat for environmental reasons | |
| No, I didn't reduce red meat consumption, nor do I intend to do it due to environmental reasons | |

6. With which protein source do you replace or would you preferably replace meat at meals?

| | YES | NO |
|---|---|---|
| Fish | | |
| Eggs | | |
| Dairy products | | |
| Protein powder (Instant meat substitutes) | | |
| Legumes | | |
| Nuts | | |
| Seitan | | |
| Tempeh | | |
| Algae | | |
| Jellyfish | | |

7. In the future, would you be willing to replace meat with each of the following food items?

| | YES | NO | I DO NOT KNOW | YES, IF TO MY TEST |
|---|---|---|---|---|
| Insects and insect derivates | | | | |
| Lab-grown meat (from cell culture) | | | | |
| Plant-based meat alternatives, only made from ingredients that are not derived from Genetically Modified Organisms (GMO) | | | | |
| Plant-based meat alternatives, even if made from ingredients derived from Genetically Modified Organisms (GMO) | | | | |

8. To what extent do you agree with each of the following statements?
[answer from 1 (strongly disagree) to 10 (strongly agree)]

| | |
|---|---|
| Eating meat is necessary to have a complete diet | |
| I need meat to have energy | |
| Eating meat allows me to have a balanced diet | |
| Meat is irreplaceable in my diet | |
| Replacing meat with plant-based protein sources doesn't provide me with the same energy | |

9. If all meat products comply with farm animal welfare rules and prices were to increase by 30% compared to current prices (Tick only one answer):

| | |
|---|---|
| I would eat the same amount of meat products as I currently do | |
| I would eat more meat products than I currently do | |
| I would eat less meat products than I currently do | |

10. To what extent do you agree that companies use meat-related names like 'sausage' and 'burger' to describe meat-free vegetarian products (e.g. a veggie 'burger')? (Tick only one answer):

| | |
|---|---|
| It should never be allowed for vegetarian products | |
| It should be allowed only if it is clearly labelled it's a vegetarian product | |
| I don't see any problem for using such names | |
| I have no opinion | |

11. To what extent do you agree with each of the following statements?
[answer from 1 (strongly disagree) to 10 (strongly agree)]

| | |
|---|---|
| Sustainability information should be compulsory on food labels | |
| Food which is less sustainable should be more taxed (and be more expensive) | |
| Unsustainable food products should be pulled from shelves (e.g. no strawberries in winter, supermarkets should only sell fish sourced sustainably, etc.) | |
| Regulations should force farmers and food producers to meet more stringent sustainability standards (in terms of greenhouse gas emissions, water use, biodiversity impact, etc.) | |
| Farmers should be given incentives (e.g. through subsidies) to produce food more sustainably | |
| The EU should not be more proactive on sustainable food policies unless other countries such as China or the USA do the same | |

12. To what extent do the following activities contribute to climate change?
[answer from 1 (strongly disagree) to 10 (strongly agree)]

| | |
|---|---|
| Emissions from aircraft, trains, cars, trucks and ships | |
| The production of meat and dairy products, which we eat and drink | |
| The felling of trees and forests | |
