# Supplementary material for: Consumers’ Attitude towards Sustainability in Italy: Process of Validation of a Duly Designed Questionnaire
Source: Foods. 2022 Aug 30;11(17):2629. doi: 10.3390/foods11172629 (PMC9455261; doi:10.3390/foods11172629)
Supplement: Supplementary file 1 [file foods-11-02629-s001.zip › Table S4.pdf]

Table S4. Reports all seven factors loadings after Varimax rotation for all questions.

Rotated Factor Pattern							
**QUESTION**	**Factor1**	**Factor2**	**Factor3**	**Factor4**	**Factor5**	**Factor6**	**Factor7**
Q1- option1	-1	12	13	-3	50*	-10	4
Q1 - option2	33*	10	15	17	-26	30*	-28
Q6 - option1	-5	52*	57*	-2	10	13	1
Q6 - option2	-8	24	85*	-6	13	-6	0
Q6 - option3	-16	17	90*	-2	-1	-4	-4
Q6 - option4	-47*	22	48*	41	21	6	7
Q6 - option5	-31*	32	40*	20	18	-6	-16
Q6 - option8	-1	-5	2	5	15	-24	43*
Q6 - option9	-1	-15	-4	-5	-9	60*	-6
Q10 - option1	85*	-2	-9	26	-1	19	-16
Q10 - option2	76*	-8	-14	-17	19	14	0
Q10 - option3	89*	-6	-4	23	-2	-1	-9
Q10 - option4	58*	-9	-6	-5	-14	-13	-7
Q10 - option6	74*	-13	-11	3	-6	-5	3
Q13 - option1	14	78*	35*	16	10	-8	29
Q13 - option2	-24	66*	0	7	33*	-11	10
Q13 - option3	-22	69*	13	-33*	19	-3	-19
Q13 - option4	27	10	-5	34*	-6	-9	-7
Q13 - option5	-19	75*	23	8	-8	-17	-12
Q13 - option6	12	76*	41*	24	-11	2	17
Q13 - option7	18	-16	5	10	8	-9	-38*

The factor loading shown in Table S4 has been colored. Printed values are multiplied by 100 and rounded to the nearest integer. Absolute values greater than 0.295614 are flagged by an '*'.
